# Side effects and complications of intraosseous anesthesia and conventional oral anesthesia

**DOI:** 10.4317/medoral.17512

**Published:** 2011-12-06

**Authors:** David Peñarrocha-Oltra, Javier Ata-Ali, María J. Oltra-Moscardó, María Peñarrocha-Diago, Miguel Peñarrocha

**Affiliations:** 1 DDS, Resident of the Master in Oral Surgery and Implantology. Valencia University Medical and Dental School; 2 DDS, Master in Oral Surgery and Medicine. Master in Oral Surgery and Implantology. Valencia University Medical and Dental School; 3 MD, DDS, PhD. Valencia University Medical and Dental School; 4 Associate Professor of Oral Surgery. Valencia University Medical and Dental School; 5 Chairman of Oral Surgery. Director of the Master in Oral Surgery and Implantology. Valencia University Medical and Dental School. Valencia (Spain)

## Abstract

Objective: To analyze the side effects and complications following intraosseous anesthesia (IA), comparing them with those of the conventional oral anesthesia techniques. Material and method: A simple-blind, prospective clinical study was carried out. Each patient underwent two anesthetic techniques: conventional (local infiltration and locoregional anesthetic block) and intraosseous, for respective dental operations. In order to allow comparison of IA versus conventional anesthesia, the two operations were similar and affected the same two teeth in opposite quadrants. Heart rate was recorded in all cases before injection of the anesthetic solution and again 30 seconds after injection. The complications observed after anesthetic administration were recorded. Results: A total of 200 oral anesthetic procedures were carried out in 100 patients. Both IA and conventional anesthesia resulted in a significant increase in heart rate, though the increase was greater with the latter technique. Incidents were infrequent with either anesthetic technique, with no significant differences between them. Regarding the complications, there were significant differences in pain at the injection site, with more intense pain in the case of IA (x2=3.532, p=0.030, Φ2=0.02), while the limitation of oral aperture was more pronounced with conventional anesthesia (x2=5.128, p<0.05, Φ2=0.014). Post-anesthetic biting showed no significant differences (x2=4.082, p=0.121, Φ2=0.009). Conclusions: Both anesthetic techniques significantly increased heart rate, and IA caused comparatively more pain at the injection site, while limited oral aperture was more frequent with conventional anesthesia. Post-anesthetic biting showed no significant differences between the two techniques.

** Key words:**Intraosseous anesthesia, oral anesthesia, mandibular block, heart rate, adrenalin, complications.

## Introduction

Intraosseous anesthesia (IA) allows direct placement of the anesthetic solution in the cancellous bone adjacent to the tooth programmed for anesthesia. Since the anesthetic solution is targeted directly to the tooth requiring treatment, the surrounding soft tissues are usually not affected (1).

In the year 2002 the American Dental Association (ADA) accepted the Stabident® system as an effective and safe technique for intraosseous pulp anesthesia, either as a primary procedure or as a complement to other anesthetic maneuvers. Many studies have been published on this intraosseous anesthesia technique (2-12). A number of authors have found IA to be associated with an increase in patient heart rate when the anesthetic solution contains adrenalin or levonordefrin. The increase in heart rate varies from 8-32 beats per minute (3,4,13-18). The administration of local anesthetics associated to adrenalin can have serious repercussions, particularly in patients who are using tricyclic antidepressants and nonselective beta-blockers. Their use in patients with cardiovascular diseases is subject to controversy (19,20).

The present study analyzes the side effects and complications of intraosseous anesthesia, and compares them with those of conventional oral anesthesia.

## Material and Methods

A simple-blind, prospective clinical study was carried out. A total of 200 oral anesthetic procedures were carried out in 100 patients. Each patient was subjected to both anesthetic techniques: conventional (local infiltration and locoregional anesthetic block) and intraosseous, for respective dental operations. A 7-day interval was established between the two procedures. Anesthesia in all cases was carried out by the same operator (Oltra-Moscardó, M.J.). In order to allow comparison of IA versus conventional anesthesia, the two operations were similar and affected the same two teeth in opposite quadrants.

Dental treatment comprised silver amalgam or composite reconstructions and root canal treatments of teeth with vital pulp tissue. In all cases attempts were made to ensure that the treatments on both sides of the mouth were the same.

The following inclusion criteria were established: patients between 10-55 years of age, the absence of disease antecedents (diabetes, heart disease, high blood pressure), the absence of medication, the absence of oral or soft tissue infections, and the confirmation of pulp vitality using thermal and electrical tests. The exclusion criteria were: periodontal (periodontal pockets or tooth mobility) or radiological alterations (bone loss or periapical radiotransparencies), as well as any type of third molar treatment.

All parents gave written informed consent to inclusion in the study, which was carried out according to the Declaration of Helsinki, following approval of the local Ethics Committee.

Heart rate was recorded in all cases before injection of the anesthetic solution and again 30 seconds after injection. The side effects and complications observed after anesthetic administration were recorded both during the dental procedure and 7 days after surgery. Discomfort and/or problems opening or closing the mouth, as well as post-anesthetic biting problems were recorded subjectively by questioning the patient 7 days after the procedure.

For conventional anesthesia we used the Aspiject® syringe (Laboratorios Inibsa, Barcelona, Spain) with an auto-aspirating system and a 25-mm injection needle. IA in turn was carried out using the Stabident® system (Fairfax Dental Inc., Miami, FL, USA), following the technique described by Gallatin et al. (4). The anesthetic solution used in the conventional procedures was 2% lidocaine with 1:100,000 adrenalin (Octocaine 1:100,000, Laboratorios Clarben, S.A., Madrid, Spain), while IA was carried out using 3% mepivacaine without vasoconstrictor (Laboratorios Normon, Normon, Madrid, Spain).

The chi-squared test was used for comparing qualitative variables. The strength of the correlation between categorical variables was evaluated using the phi-square coefficient (Φ). The parametric Student t-test in turn was used to assess differences between the means of two groups. Comparison between the two anesthetic techniques in one same patient was carried out using the McNemar test. Statistical significance was accepted for p≤0.05.

## Results

The mean patient age was 28.6 ± 9.92 years (range 11-55); there were 47 males and 53 females. In relation to the conventional anesthetic technique, 55 vestibular infiltrations (51 in the upper maxilla, and 4 in lower incisors and canines) and 45 mandibular blocks were carried out. All 100 patients were also subjected to IA (51 in the upper maxilla, and 49 in the mandible).

In the case of conventional anesthesia, three patients (3%) received half a carpule, 78 patients (78%) received one carpule, 18 patients (18%) received two carpules, and one patient (1%) received more than two anesthetic carpules. In all cases 2% lidocaine with 1:100,000 adrenalin was used, with an average of 1.18 anesthetic carpules per treatment. In the case of intraosseous anesthesia, 91 patients (91%) received half a carpule or less, while 9 patients (9%) received one carpule. In these patients we always used 3% mepivacaine without vasoconstrictor, with an average of 0.55 anesthetic carpules per treatment. It is thus seen that conventional anesthesia required larger volumes of anesthetic solution than IA.

Blood aspiration was observed in 5% of the operations with conventional anesthesia (4 inferior alveolar nerve blocks and one infiltrating anesthetic procedure) and in 61% of the IA techniques – the difference between the two groups being significant (x2=70.92, p≤0.0000, Φ2=0.36).

Both IA and conventional anesthesia resulted in a significant increase in heart rate, though the increase was greater with the latter technique. The heart rate values are described in ( Table 1).

**Table 1:**
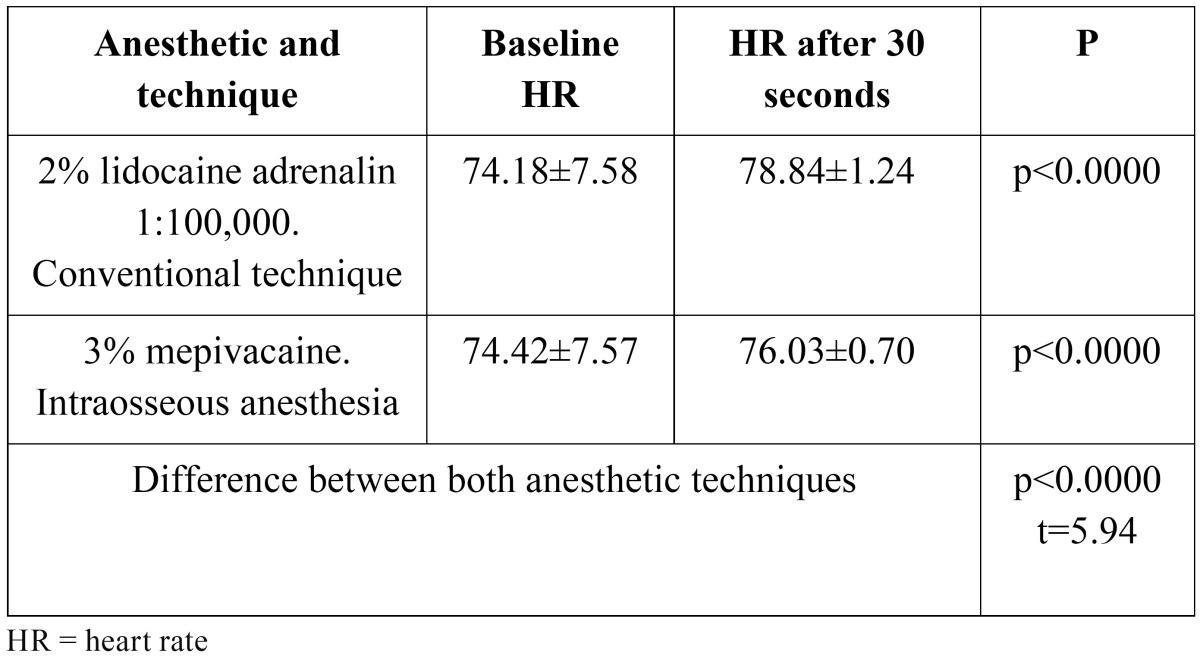
Summary of heart rate at baseline and 30 seconds after administration of the anesthetic solution.

Incidents were infrequent with either anesthetic technique, with no significant differences between them. Three patients (3%) subjected to conventional anesthesia and 7 patients (7%) subjected to IA reported dizziness, weakness in the legs and slight perspiration after injection. In the conventional group, a close correlation was found between dizziness as an immediate side effect of anesthesia and positive blood aspiration (x2=24.76, p≤0.0000, Φ2=0.25). Perforation proved difficult in 5 cases with IA, and in 6 cases we had problems finding the perforation orifice – a new maneuver being necessary in such cases (double perforation).

Regarding the complications, there were significant differences in pain at the injection site, with more intense pain in the case of IA (x2=3.532, p=0.030, Φ2=0.02), while the limitation of oral aperture was more pronounced with conventional anesthesia (x2=5.128, p<0.05, Φ2=0.014). Post-anesthetic biting showed no significant differences (x2=4.082, p=0.121, Φ2=0.009). The patients with such complications are summarized in ( Table 2).

**Table 2:**
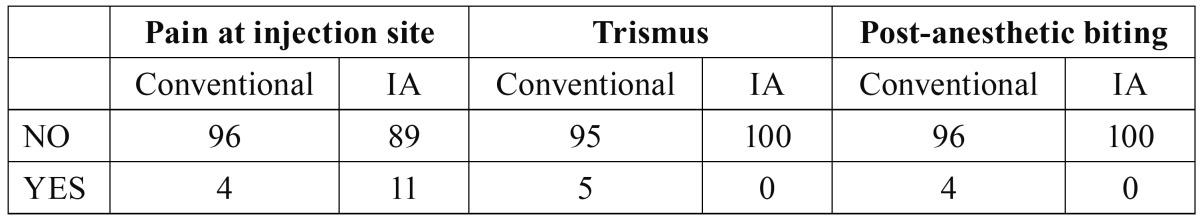
Summary of patients with pain at the injection site, discomfort at oral aperture (trismus), and post-anesthetic biting problems.

## Discussion

One of the many advantages of intraosseous anesthesia (IA) is the possibility of performing bilateral mandibular anesthesia, due to the absence of anesthesia of the lip and tongue. An additional advantage is the use of a lesser volume of anesthetic solution (5). Furthermore, if repeat anesthesia proves necessary, the anesthetic solution can be added through the already produced perforation (21). Although the Stabident manual recommends 0.9 ml of anesthetic solution in order to anesthetize three adjacent teeth, Replogle et al. (12) used 1.8 ml, while the average volume in our study was 0.99 ml.

In the present study, 5% of the patients subjected to conventional anesthesia showed blood aspiration. This percentage is similar to that reported in a series of 143 patients (4.3%)(22). In the conventional group, a significant correlation was found between dizziness as an immediate side effect of anesthesia and positive blood aspiration – though aspiration showed that these situations did not correspond to intravascular injection of the anesthetic solution. Positive blood aspiration in turn was recorded in 61% of the IA cases, and theoretically with this anesthetic technique aspiration should prove positive in all patients, since the needle was positioned in bone marrow. However, the scant negative pressure exerted may have been responsible for the observed 39% negative aspirations rate. After positioning the needle in the drilled foramen, some authors (2,12) describe that if much pressure must be applied to the plunger, they twist the syringe and needle and again attempt to inject the anesthetic solution. If this fails, they extract the needle to check whether it has become obstructed; if not, a new perforation is made. It is important to apply negative pressure by aspirating, since we very possibly may find the tip of the needle to be lodged in cancellous bone, with no obstruction of the needle – and thus the required pressure will be very gentle.

The literature describes that IA with vasoconstrictor raises the patient heart rate (3,4,13-18). In addition, as early as 1985, Rawson and Orr (23) found vascular penetrability of the anesthetic in IA to elicit a systemic effect similar to that associated with an intravascular injection. This is the reason why we decided not to add adrenalin to IA in the present study, with a view to avoiding its direct effect upon heart rate. Clinically this may represent an advantage, though we found the heart rate to increase despite the absence of vasoconstrictor. While a transient increase in heart rate in healthy patients is not important, it may prove relevant in medically compromised patients or in individuals receiving medication in which associated vasoconstrictor use is contraindicated. In such patients 3% mepivacaine is a good choice (10,18,24,25). The literature contains many studies that analyze the effects of the vasoconstrictors added to local anesthetic solutions upon patient heart rate (4,17,18,26), though such effects are not due only to the associated vasoconstrictor agent and can also be attributed to the pharmacological action of the anesthetic itself. Ezmek et al. (27), in a study of 60 patients, found 3% mepivacaine and 2% lidocaine (both without vasoconstrictor) to significantly increase heart rate. These results coincide with our own observations, where both IA with 3% mepivacaine and conventional anesthesia with 2% lidocaine plus adrenalin 1:100,000 induced a significant increase in heart rate. As suggested by Liau et al. (28), this may be due to the release of endogenous adrenalin secondary to patient emotional stress – not to the effect of the local anesthetic. However, other studies (13,24) have not found IA with 3% mepivacaine to increase heart rate.

There were no important incidents in our study. It should be mentioned that 7 patients subjected to IA and three patients receiving conventional oral anesthesia suffered dizziness, weakness in the legs, mild perspiration and tachycardia. The comparatively greater frequency of these manifestations in the IA group possibly could be due to the rapid penetration of the anesthetic into the bloodstream, vasovagal reactions of psychic origin, or reactions to the slight pain produced by the injection. As regards the need for double perforation in cases where the first point of penetration cannot be located, a study of 42 patients (12) found the repetition of perforation to be necessary in 9% of the cases (versus in 6% in our series). Therefore, on the basis of our experience, we recommend placing special attention on the cortical perforation angle, in order to reproduce it precisely during penetration of the needle and thus avoid this complication.

Both anesthetic techniques significantly increased heart rate, and IA caused comparatively more pain at the injection site, while limited oral aperture was more frequent with conventional anesthesia. Post-anesthetic biting showed no significant differences between the two techniques.
